# Responses to drought stress among sex morphs of *Oxyria sinensis* (Polygonaceae), a subdioecious perennial herb native to the East Himalayas

**DOI:** 10.1002/ece3.1178

**Published:** 2014-10-03

**Authors:** Jie Yang, Lijuan Hu, Zhengkun Wang, Wanlong Zhu, Lihua Meng

**Affiliations:** 1 School of Life Sciences, Engineering Research Center of Sustainable Development and Utilization of Biomass Energy Ministry of Education, Key Laboratory of Yunnan for Biomass Energy and Biotechnology of Environment, Yunnan Normal UniversityKunming, 650500, China; 2 Jinan Municipal Bureau of Landscape and ForestryJinan, 250033, China

**Keywords:** Dioecy, drought stress, Hengduan Mountains, physiological response, xerothermic river valley

## Abstract

It is generally accepted that dioecious plants occur more frequently in dry and nutrient-poor habitats, suggesting that abiotic stress factors could contribute to evolution of dioecy from hermaphrodite. Therefore, experimental investigations on the responses of subdioecious species, a special sexual system comprising male, female, and hermaphrodite plants, to abiotic stress factors could quantify the contribution of selective pressure on the evolution of dioecy. In this study, we evaluated the physiological responses of different sex morphs of *Oxyria sinensis* Hemsley, a perennial herb native to the East Himalayas, to drought stress. Male, female, and hermaphrodite plants of *O. sinensis* were subjected to low, moderate, and high drought stress conditions in a glasshouse. Generally, with increasing water stress, the values of most measured variables slightly decreased, whereas water-use efficiency slightly increased. Furthermore, there were no significant differences in most of the measured parameters among the sex morphs under each drought stress treatment, indicating that *O. sinensis* might be well-adapted to drought stress conditions as its typical habitat is the dry and hot habitats of xerothermic river valleys. However, nitrogen-use efficiency was significantly higher in male and female plants than in hermaphrodite plants under high drought stress conditions, suggesting that that nitrogen-use efficiency under conditions of drought stress might have contributed to the evolution of dioecy from the hermaphrodite to some degree.

## Introduction

Plants are exposed to biotic and abiotic stresses with varying degrees of severity throughout their lifetime. Water deficit is one of the most important abiotic stresses, and it can have severe negative effects on plant growth, reproduction, and production (Bartels and Sunkar 2005). When plants are exposed to drought, there are several general reactions, such as stomatal closure, decreased gas exchange rates, and accumulation of osmolytes (Chaves et al. 2009). Unisexual plants often show sex-specific characters, which may result from different resource constraints on sexual functions and the ecological differentiation of male and female plants. It is generally accepted that female plants often invest more into reproduction than do male plants (e.g., Cipollini and Whigham 1994; Bochenek and Eriksen 2010; but see Zhao and Yang 2008). Regarding the different responses of dioecious plants to drought, females are considered to be more sensitive than males to drought stress (Li et al. 2004; Xu et al. 2008; Chen et al. 2010), which might result from the higher proportional investment into reproduction in females than in males (Garcia and Antor 1995; Bochenek and Eriksen 2010). A strong association was found between harsh abiotic environments and the occurrence of sexual dimorphism (Ashman 2006), suggesting that abiotic environmental stresses might have contributed to the evolution of dioecy. However, this hypothesis has rarely been tested experimentally.

Generally, for the gynodioecious species in dry or nutrient-poor habitats, hermaphrodites often produced fewer seeds than female plants (Ashman 2006), indicating that hermaphrodites in low-resources populations may be genetically more male than those in high-resources sites because female frequency would increase with resource decline and the high frequencies of females would select for the increased maleness of hermaphrodites (Charlesworth 1989). Therefore, it would be reasonable to speculate that dry habitats might contribute to the evolution of dioecy, but it is still unclear whether there is a differentiation of drought tolerance between hermaphrodite and dioecious plants because almost no research has been performed to quantify the ability of drought tolerance of hermaphroditic and dioecious plants. Subdioecy, in which there are male, female, and hermaphrodite plants of a species, is generally considered to be an intermediate phase between cosexuality and dioecy (Barrett 1992), although there is little experimental evidence supporting this idea. Therefore, by employing the subdioecious species, examinations on the different responses to drought stress of hermaphroditic and dioecious plants would specifically help us to understand the role of abiotic factors on the evolution of dioecy.

*Oxyria*, a small genus in the Polygonaceae, is composed of two species, *Oxyria digyna* (L.) Hill and *Oxyria sinensi*s Hemsley (Sun et al. 2012), reported as hermaphroditic and dioecious species, respectively (Li et al. 2003). Nevertheless, *O. sinensis*, has both hermaphroditic and dioecious plants that coexist in some populations, as determined during our field investigations, but most of the populations we evaluated consisted of male and female plants only. Flowers of *O. digyna* are hermaphroditic, and thus, the dioecious plants of *O. sinensis* should be a derived phase (Barrett 1992). Collectively, considering the wide distribution and the derived the stage of dioecious plants of *O. sinensis* and their typical habitats in the xerothermic valleys of the Hengduan Mountains, we hypothesized that hermaphroditic plants of *O. sinensis* might be less tolerant than dioecious plants to drought stress. If this is the case, the differences in drought tolerance may contribute to the evolution of dioecy in *O. sinensis* to some degree (e.g., Hart 1985; Weller and Sakai 2005). To test our hypothesis, we subjected hermaphroditic and dioecious plants of *O. sinensis* to different soil water content treatments and analyzed the drought tolerance of the sex morphs. Specifically, our aims were as follows: (1) to determine whether there are differences in drought tolerance between hermaphroditic and dioecious plants; and (2) to determine whether there are differences in drought tolerance between male and female plants.

## Material and Methods

### Plant materials and experimental design

**Figure 1:**
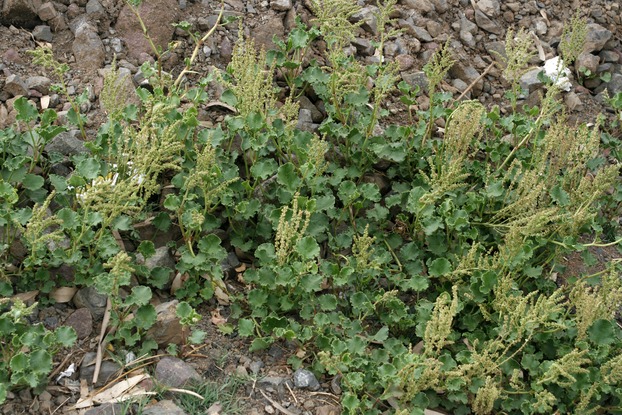
Plants and the habitat of *Oxyria sinensis*.

*Oxyria sinensis* is a perennial herb native to the East Himalayas (Fig. 1). It can reproduce sexually via seeds and asexually via rhizomes (Zhao and Yang 2008). This plant is characterized by tiny flowers, densely branched panicles, and winged achenes that might favor wind dispersal. In the Hengduan Mountains, *O. sinensis* is abundantly distributed along the roadsides and in abandoned farmlands along the riversides at altitudes below 3800 m, in xerothermic valleys. Most populations of *O. sinensis* consist of male and female plants only, but in some populations, especially those along the Jinshajiang River (the upper Yangtze River), there are some hermaphroditic plants. Furthermore, the hermaphroditic and dioecious plants were mixed and occupied the same habitats in these populations.

The seeds of *O. sinensis* are easily collected in the field, but their germination rate was very low in our preliminary experiments. Therefore, we used rhizomes to cultivate experimental plants. We collected rhizomes 10 cm in length from field populations containing male, female, and hermaphroditic plants in April, 2013. The rhizomes were then brought to the experimental site at Yunnan Normal University, Kunming, Yunnan Province. The rhizomes were grown into plants in a canopied and naturally lit glasshouse. The sides of the glasshouse were always opened for aeration throughout the experiment, so that the temperature inside the glasshouse was closely linked to the ambient outside temperature. In the glasshouse, the rhizomes were planted in 90 pots containing a homogeneous mixture of equal volumes of peat and perlite. Ten “empty” pots were filled with the same amount of the soil mixture, but without seedlings, to measure evaporation rates. Each pot was 24 cm high with upper and lower diameters of 21 cm and 17 cm, respectively. All pots were filled with the same weight of a homogeneous soil mixture (1.32 kg), and the soil water content of each pot was completed to maximum field capacity (FC) by adding 2.95 kg water. All pots were periodically watered to maximum FC for 2 months after repotting to allow the seedlings to become established.

The experimental period commenced on July 8, 2013, and continued until August 23, 2013. Thirty plants of each sex morph were subjected to each of the low, moderate, and high drought stress treatments, each group comprising 10 plants. In each treatment, five plants were used for photosynthesis measurements and the other five plants were used for carbon isotope composition and leaf elemental analyses. The different levels of drought stress (low, medium, and high) were imposed by watering to 80%, 50%, and 20% of maximum FC, respectively. Soil water content was maintained at these levels by weighing the pots every 2 days, recording the amount of water lost, and then immediately rewatering to reach the desired water content.

### Measurements of leaf photosynthesis, transpiration, and chlorophyll fluorescence

Maximum photosynthetic rate (*A*_max_), stomatal conductance (*g*), and transpiration (*E*) were measured using a LI-COR 6400XT infrared gas analyzer (IRGA; LI-COR Biosciences, Lincoln, NE). Measurements were taken between 11:00 and 14:00 h on a sunny day (August 24, 2013) from five plants in each treatment for each sex morph. Light levels were maintained at 1200 *μ*mol·m^−2^·s^−1^, which was higher than the light-saturation point of all three sex morphs (light-saturation points were derived from light response curves determined before the experiment). Light was provided by an LI-6400-02B LED lamp (LI-COR Biosciences). The external CO_2_ concentration was maintained at 400 *μ*mol·mol^−1^ using portable tanks containing a CO_2_/air mixture. The output of the tanks was controlled by a LI-6400-01 CO_2_ injector (LI-COR Biosciences). Temperature and relative humidity were maintained as 24–26°C and 23–29%, respectively. Instantaneous water-use efficiency (WUE_*i*_) was calculated and defined as *A*_max_/*E*.

Chlorophyll fluorescence parameters were measured between (0500 and 0600 h) on leaves that had been dark adapted for 30 min. These measurements were taken on the same day as the leaf gas exchange measurements. Maximum quantum yield of photosystem II (PSII) (*F*_*v*_/*F*_*m*_ = (*F*_*m*_ − *F*_*o*_) / *F*_*m*_) was measured using a LI-6400-40 leaf chamber fluorometer (LI-COR Biosciences). Five plants in each treatment per sex morph were analyzed.

### Carbon isotope composition and leaf elemental analysis

Carbon isotope composition (*δ*^13^C) has been shown to serve as a proxy for integrated water-use efficiency (Jones 1993; Zhang and Marshall 1994; Osorio et al. 1998). Thus, to evaluate the differences in the integrated water-use efficiency among the three sex morphs, we measured the carbon isotope composition of the leaves from wild plants and the treated plants under different drought stress treatments. In the laboratory, leaves with a dry weight of approximately 0.2 g were collected and then finely ground with a Tissuelyzer (Retsch, Haan, Germany). Each sample was divided into two subsets. One subset was used to analyze nitrogen and carbon contents of the needles with a CHN analyzer (Vario EL; Elementar, Hanau, Germany). The nitrogen-use efficiency (NUE), defined as the amount of organic matter produced per unit of nitrogen taken up (Vitousek 1982), was calculated as the reciprocal of the nitrogen content. The second subset was used to analyze the carbon isotope composition of leaves (*δ*^13^C). The samples were combusted in an elemental analyzer EA1108 (Carlo Erba, Milan, Italy) and analyzed using a Finnigan Delta Plus isotope mass spectrometer (Thermo Finnigan MAT GmbH, Bremen, Germany). Carbon isotope composition was calculated relative to the Pee Dee Belemnite (PDB) standard as the ratio (‰): *δ*^13^C = (*R*_sample_/*R*_standard_ − 1) × 1000, where *R*_sample_ and *R*_standard_ are the ratios of ^13^C:^12^C in the sample and the standard, respectively. All measurements were performed at the Key Laboratory of Plateau Lake Ecology and Global Change, Yunnan Normal University, China.

### Statistical analyses

Data for all measured variables were analyzed by the general linear model (Proc GLM) to test the effects of different species, different drought treatments, and the interactions between them. Significant differences among sex morphs in a particular treatment, or among treatments for a particular sex morph, were compared using one-way analysis of variance (ANOVA). The homogeneity of variances was tested before performing ANOVA. All statistical analyses were carried out using the SPSS statistical software package.

## Results

### Leaf photosynthesis and chlorophyll fluorescence

All the measured variables were significantly affected by the watering treatment, but not by sex, or by the treatment × sex interaction (Table 1). Generally, there were no significant differences among the sex morphs in each drought stress treatment for most of the measured variables, including *A*_max_, *g*, *E*, WUE_*i*_, and *F*_*v*_/*F*_*m*_ (Tables 2), except for *A*_max_ of different sex morphs subjected to 20% FC (Table 2).

**Table 1:** Measured parameters for male, female, and hermaphrodite plants of *Oxyria sinensis*, with degrees of freedom in the brackets.

Parameter	Abbrev.	Watering treatment	Sex	Treatment × Sex interaction
Maximal rate of photosynthesis	*A*_max_	5.91** (2)	0.82 (2)	0.95 (4)
Stomatal conductance	*g*	8.08** (2)	1.60 (2)	0.45 (4)
Transpiration	*E*	6.08** (2)	2.17 (2)	0.45 (4)
Instantaneous water-use efficiency	WUE_*i*_	4.19** (2)	1.18 (2)	0.04 (4)
Maximum quantum yield of PS II	*F*_*v*_/*F*_*m*_	4.63* (2)	0.54 (2)	2.09 (4)
Carbon content	C%	1.70 (2)	3.16 (2)	0.77 (4)
Nitrogen-use efficiency	NUE	1.84 (2)	4.07* (2)	1.20 (4)
Carbon isotope composition	*δ*^13^C	18.08** (2)	0.24 (2)	1.73 (4)

Significance is shown for watering treatment, sex morph, and their interactions.

* *P* < 0.05;

** *P* < 0.01.

**Table 2:** Comparison of measured parameters among three sex morphs of *Oxyria sinensis* under three different drought treatments (soil water at 80% of maximal field capacity (FC), 50% FC, and 20% FC).

	Drought treatments (% of maximum FC)		
	Low stress	Moderate stress	High stress
Variable and species	80% FC	50% FC	20% FC
Maximal rate of photosynthesis (*A*_max_) (*μ*mol m^−2^ s^−1^)			
Male	15.41 ± 0.94 A,X	15.02 ± 1.56 A,X	11.16 ± 0.83 A,Y
Female	13.51 ± 0.99 A,XY	14.31 ± 0.45 A,X	11.79 ± 0.88 AB,Y
Hermaphrodite	14.32 ± 0.85 A,X	14.74 ± 1.38 A,X	13.58 ± 0.26 B,X
Stomatal conductance (g) (mmol m^−2^ s^−1^)			
Male	0.53 ± 0.11 A,X	0.61 ± 0.12 A,X	0.25 ± 0.04 A,Y
Female	0.36 ± 0.09 A,X	0.46 ± 0.02 A,X	0.28 ± 0.05 A,X
Hermaphrodite	0.50 ± 0.12 A,XY	0.62 ± 0.09 A,X	0.34 ± 0.04 A,Y
Transpiration (E) (mmol m^−2^ s^−1^)			
Male	3.72 ± 0.47 A,XY	4.40 ± 0.37 A,X	2.80 ± 0.35 A,Y
Female	3.11 ± 0.52 A,X	3.96 ± 0.11 A,X	2.99 ± 0.44 A,X
Hermaphrodite	3.76 ± 0.54 A,X	4.59 ± 0.38 A,X	3.78 ± 0.31 A,X
Instantaneous water-use efficiency (WUE_*i*_) (*A*_max_/*E*)			
Male	4.36 ± 0.49 A,X	3.42 ± 0.28 A,X	4.21 ± 0.50 A,X
Female	4.62 ± 0.44 A,X	3.61 ± 0.11 A,X	4.26 ± 0.56 A,X
Hermaphrodite	4.08 ± 0.49 A,X	3.21 ± 0.11 A,X	3.71 ± 0.36 A,X
Maximum quantum yield of PS II (*F*_*v*_/*F*_*m*_)			
Male	0.82 ± 0.007 A,X	0.83 ± 0.002 A,X	0.83 ± 0.002 A,X
Female	0.82 ± 0.004 A,X	0.83 ± 0.002 A,X	0.83 ± 0.004 A,X
Hermaphrodite	0.83 ± 0.005 A,X	0.83 ± 0.003 A,X	0.83 ± 0.005 A,X

Values are mean ± SE.

Different letters after values indicate significant differences (*P* < 0.05) among different drought treatments (X, Y, Z) and different sex morphs (A, B).

The value of *A*_max_ decreased significantly under high drought stress in male and female plants, but not in hermaphrodite plants. The values of *g* and *E* decreased significantly under high drought stress only in male plants (Table 2). There were no significant differences in WUE_*i*_ or *F*_*v*_/*F*_*m*_ among the drought stress treatments for each sex morph (Table 2).

### Nitrogen-use efficiency and carbon content

**Figure 2:**
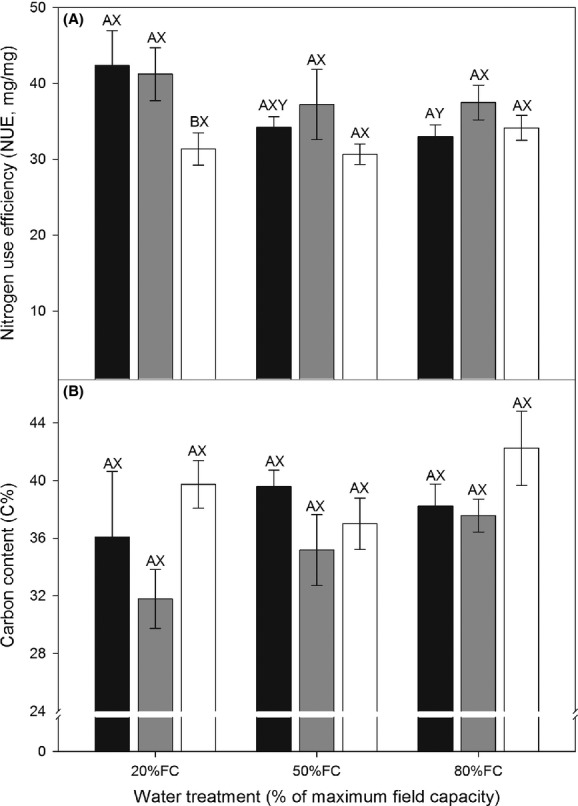
(A) Nitrogen-use efficiency (NUE) and (B) carbon content (C%) in leaves of male (black bars), female (gray bars), and hermaphrodite (open bars) plants of *Oxyria sinensis* under different drought conditions (soil water at 80% of maximal field capacity (FC), 50% FC, 20% FC). Data are means + SE. Different letters indicate significant differences (*P* < 0.05) among different drought treatments (X, Y) and different sex morphs (A, B).

Overall, the NUE was significantly different between sex morphs, but not affected significantly by the drought treatment or the drought treatment × sex interaction (Table 1). Under 20% FC, the NUE was significantly lower in hermaphrodite plants than in male and female plants, but there were no significant differences among the three sex morphs under 50% FC or 80% FC (Fig. 2A). In addition, the NUE of male plants was higher under 20% FC, but there was no significant difference in the NUE of female and hermaphrodite plants among the three drought treatments (Fig. 2A). Carbon concentration (C%) was not significantly affected by drought treatment, sex morph, or the drought treatment × sex morph interaction (Table 1). Thus, there was no significant difference in C% among sex morphs in each drought treatment and among drought treatments for each sex morph (Fig. 2B).

### Carbon isotope composition (δ^13^C)

**Figure 3:**
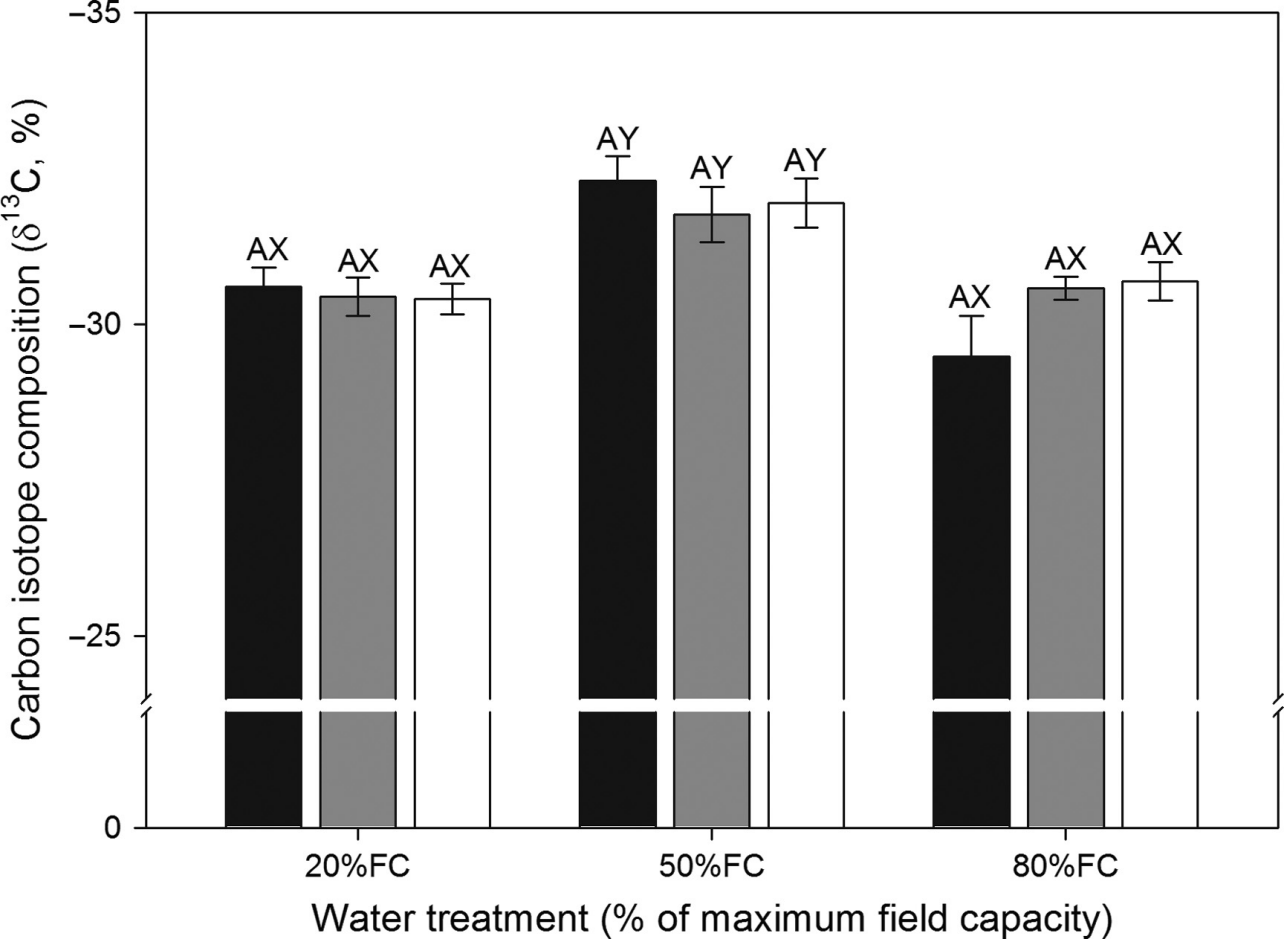
Carbon isotope compositions (*δ*^13^C) of leaves of male (black bars), female (gray bars), and hermaphrodite (open bars) plants of *Oxyria sinensis* under different drought conditions (soil water at 80% of maximal field capacity (FC), 50% FC, 20% FC). Data are means + SE. There were no significant differences among different drought treatments and different sex morphs.

For each sex morph, the amount of *δ*^13^C was significantly higher under 20% FC and 80% FC than under 50% FC. There was no significant difference in *δ*^13^C among sex morphs across all drought treatments (Fig. 3). Collectively, *δ*^13^C was affected significantly by drought treatment, but not by sex morph and or the drought treatment × sex morph interaction.

## Discussion

### Ecophysiological responses of *Oxyria sinensis* to drought stress

Water availability is one of the most important factors constraining plant growth and distribution (Kramer and Boyer 1995). Thus, plants growing in water-limited regions have developed various mechanisms to tolerate drought. Such mechanisms include adjustments of physiological and morphological characteristics, such as changes in structure, water-use efficiency, and stomatal conductance (Kozlowski and Pallardy 2002). We observed similar patterns in *O. sinensis*. In the present study, high drought stress (20% FC) resulted in decreases in *A*_max_, *g*, and *E* in each of the sex morphs, although the differences were not significant across all variables. Under drought stress, *g* is believed to be more sensitive than other parameters (Schulze et al. 1998); this is consistent with the results for each sex morph of *O. sinensis* obtained in the present study. Under moderate drought stress conditions, drought-stressed male, female, and hermaphrodite plants showed 59, 39, and 45% lower values of *g*, respectively, but only 26, 18, and 8% lower values of *A*_max_, respectively, compared with their respective values under low drought stress conditions (Table 2). Changes in stomatal conductance may contribute to similar decreases in transpiration rates under increasing drought stress. The male, female, and hermaphrodite plants showed 26, 24, and 18% reductions in transpiration rates, respectively, under high drought stress relative to moderate drought stress. Greater decreases in *g* than in *A*_max_ under drought stress have also been observed in other plant species (e.g., Ismail and Hall 1992; Zhang and Marshall 1994; Ma et al. 2010).

We found no decreases in WUE_i_, *δ*^13^C, or *F*_*v*_/*F*_*m*_ under increasing drought stress, although *g* showed a decreasing trend. One possible explanation for these results might be the physiological attributes of *O. sinensis*, which usually inhabits hot, dry xerothermic valleys. Therefore, *O. sinensis* may be well-adapted to drought conditions, and thus, the level of drought stress in our experiments was not strong enough to damage PS II in this species. Therefore, the decreasing trends in *A*_*max*_ and *E* might be caused by stomatal limitation (Flexas et al. 2014), even under conditions of high drought stress.

For plant species with separate sex morphs, the various morphs might be affected differently under water stress, because male and female plants differ in mass, nutrient composition, and water content (Harris and Pannell 2008). However, few studies have focused on evaluating sex-related differences in stress tolerance in plants. The limited studies that have been conducted have suggested that reduced performance of the female under stressful environments is because of its greater investment in reproduction (Li et al. 2007; Xu et al. 2008; Rozas et al. 2009; Zhang et al. 2010), although this is not a generalized pattern (Onate and Munne-Bosch 2009). For *O. sinensis*, no significant differences were found in *A*_*max*_*, g*, and *E* between male and female plants, probably because of the habitat of this species, as mentioned above.

### Implications for the evolution of dioecy

As speculated by Darwin (1887), “a very dry station apparently favours the presence of the female form”. Thus, drought stress could be one of the drivers for the evolution from the hermaphrodite to dioecy, although the exact reason remains unclear (Ashman 2006). Considering the hermaphroditic *O. digyna*, the only congener of *O. sinensis*, male and female plants could be a derived stage. Therefore, as we have predicted, if drought stress has contributed to the evolution from the hermaphrodite to dioecy in *O. sinensis*, then hermaphrodite plants should be more sensitive than male and female plants to drought stress. However, our results suggested that only NUE under drought conditions was affected significantly by the sex morphs (Table 1). The NUE reflects the ecological fitness of plants, because nitrogen is necessary for their growth (Aerts and Chapin 2000). In our study on *O. sinensis*, under increasing water stress, the NUE increased in male and female plants but decreased in hermaphrodite plants. Also, the NUE of male and female plants was significantly higher than that of hermaphrodite plants under high drought stress conditions (Fig. 2A). Considering the dry and hot habitat of *O. sinensis*, the high NUE of male and female plants under high drought stress might have contributed to the evolution of dioecy to a certain degree. However, this species has a strong capacity for clonal growth, resulting in a high density of ramets within one ramet, and there is also potential for wind pollination (Zhao and Yang 2008). These attributes can lead to intense inbreeding. The resulting inbreeding depression in hermaphrodite plants growing in hot, dry habitats may have played a major role in driving the evolution of dioecy (Ashman 2006), although our results indicated that high NUE contributed to the evolution of dioecy to some degree.
